# Influence of rivaroxaban compared to vitamin K antagonist treatment upon development of cardiovascular calcification in patients with atrial fibrillation and/or pulmonary embolism

**DOI:** 10.1002/clc.23819

**Published:** 2022-03-25

**Authors:** Robert Stöhr, Timm Dirrichs, Kinan Kneizeh, Sebastian Reinartz, Dario Frank, Johannes Brachmann, Joerg Schroeder, Leon Schurgers, Claudia Göttsch, Andras Keszei, Jürgen Floege, Nikolaus Marx, Vincent Brandenburg, Alexander Schuh

**Affiliations:** ^1^ Department of Cardiology RWTH University Hospital Aachen Aachen Germany; ^2^ Department of Radiology RWTH University Hospital Aachen Aachen Germany; ^3^ Department of Internal Medicine St. Antonius Hospital Eschweiler Germany; ^4^ Department of Cardiology Regiomed‐Kliniken Coburg Germany; ^5^ Department of Biochemistry Maastricht University Hospital Maastricht The Netherlands; ^6^ Institute of Experimental Medicine and Systems Biology RWTH Aachen University Aachen Germany; ^7^ Center for Translational and Clinical Research RWTH Aachen University Aachen Germany; ^8^ Department of Nephrology RWTH University Hospital Aachen Aachen Germany; ^9^ Department of Cardiology and Nephrology Rhein‐Maas Klinikum Würselen Germany; ^10^ Department of Cardiology St.‐Katharinen‐Hospital Frechen Germany

**Keywords:** coronary/valvular calcification, rivaroxaban versus coumadin/phenprocoumon treatment

## Abstract

**Background:**

Vitamin K antagonists (VKA) such as warfarin or phenprocoumon have been the mainstay of therapy for long‐term oral anticoagulant therapy (OAT) in patients with atrial fibrillation or with pulmonary embolism. Due to interferences with matrix Gla‐protein, an important vitamin K‐dependent local calcification inhibitor in cardiovascular structures, VKA antagonists stimulate cardiovascular calcification (CVC). In contrast, rivaroxaban, a nonvitamin K‐dependent oral anticoagulant (NOAC), should be neutral in terms of CVC. We seek to investigate these potential differences in CVC development between VKA versus NOACs in a randomized controlled trial (RCT).

**Methods:**

The influence of rivaroxaban compared to vitamin K antagonist treatment upon development of cardiovascular calcification in patients with atrial fibrillation and/or pulmonary embolism trial (NCT02066662) is a multicenter, prospective RCT with a two‐arm, open‐label study design. The primary endpoint is the progression of coronary and aortic valve calcification (quantified as calcification volume score) as assessed by cardiac computed tomography (CT) at 24 months in patients either treated by rivaroxaban or VKA. A total of 192 patients were randomized in a 1:1 fashion. The main inclusion criteria were the presence of atrial fibrillation and/or pulmonary embolism with the indication for OAT and pre‐existent coronary calcification. The development of CVC will be assessed by follow‐up CT at 12 and 24 months.

**Results:**

In total 192 patients (median age 70, 72% male) were enrolled over a period of 5 years and followed up for 2 years.

## 1 INTRODUCTION

Atrial fibrillation is one of the most frequent indications for long‐term oral anticoagulant therapy (OAT). Long‐term OAT with vitamin K antagonists (VKA), such as warfarin or phenprocoumon, reduces the risks of stroke and death in patients with atrial fibrillation. Therefore, VKA have been the therapeutic cornerstone for patients with atrial fibrillation at increased thromboembolic risk for decades. Several well‐known drawbacks reduce the efficacy and safety of VKA. First, they increase the risk of bleeding as compared to control therapy.^1^, ^2^ Moreover, VKA have multiple food and drug interactions, and therefore, require frequent laboratory monitoring for optimal dosing. Thus, the rate of discontinuation and side effects is high and many patients receiving VKA still have inadequate anticoagulation (low time within target range regarding international normalized ratio [INR]).^3^, ^4^, ^5^ Indeed, up to half of all these patients which is also true for patients with pulmonary embolism, fail to continuously stabilize their INR within the target range resulting in increased risk for thromboembolism and/or bleeding complications.^6^

Recent years have shown a remarkable evolution in OAT with the emergence of the so‐called nonvitamin K‐dependent oral anticoagulant drugs (NOACs). The first‐in‐class representative of the NOACs was Rivaroxaban. A randomized trial in patients with atrial fibrillation (ROCKET‐AF) revealed noninferiority for Rivaroxaban compared to VKA‐based oral anticoagulation in terms of stroke prevention or systemic embolism.^3^ Similarly, a large RCT has proven noninferiority of Rivaroxaban compared to VKA in patients with deep vein thrombosis or pulmonary embolism.^7^

### 1.1 The role of vitamin antagonists in the promotion of vascular calcification

Matrix Gla‐protein (MGP), a vitamin K‐dependent protein, was identified in the human arterial wall and cartilage where the major biological function is the prevention of ectopic calcification. To be fully biologically active, MGP requires vitamin K for posttranslational modification (gamma‐carboxylation in a similar way like the clotting factors II, VII, IX, and X). These anticalcification properties were convincingly demonstrated in MGP‐knockout mice that die from massive aortic calcification (vascular “fracture”) shortly after birth.^4^

Several studies have suggested that treatment with a VKA can increase the rate of development of coronary and valvular arterial calcification,^8^, ^9^, ^10^ whereas the intake of Vitamin K was, in some studies, associated with a slowing of the progression of calcification.^5^, ^11^

Epidemiological, experimental in vivo, and noninterventional human data have supported the theory that vitamin K is essential for the physiological function and phenotype of all human tissues, which should be protected from ectopic calcification. This is especially evident in the tunica media of the vascular wall and the cartilage.^5^ Hence, an important possible side effect of VKA is induction and/or accelerated progression of coronary and valvular calcification.^6^, ^12^, ^13^ Rivaroxaban, on the other hand, inhibits Xa factor without interaction with vitamin K. The COMPASS Trial demonstrated that rivaroxaban plus aspirin compared with aspirin alone reduced the composite endpoint of cardiovascular death, myocardial infarction, or stroke.^14^

In recent years smaller prospective trials have attempted to investigate the differential effects of VKA on calcification. Win et al. found that in comparison to Rivaroxaban, a 52‐week course or warfarin increased plaque volume and calcification as measured by MultiSlice CT (MSCT).^15^ The same group showed that similar effects were obtained with apixaban after 52 weeks compared to warfarin.^16^ This may suggest that the use of a NOAC as OAT may prevent the development of coronary calcification compared to VKA, however, both studies were single centered and included less than 50 patients per group for a relatively short follow‐up period of 52 weeks. Similarly, there have been smaller studies comparing the development of valvular calcification showing a protective effect of NOAC therapy compared to VKA in murine models.^17^ A retrospective study comparing patients on VKA and NOAC also found VKA therapy to be significantly associated with faster progression rates of hemodynamic and anatomic aortic stenosis severity.^18^

In summary, there is currently some evidence from murine and smaller trials, that the use of NOACs may reduce vascular and valvular calcifications compared to VKA therapy in patients on OAT. However, most of the trials were either retrospective or included smaller numbers of patients or shorter duration of follow‐up. The influence of rivaroxaban compared to vitamin K antagonist treatment upon development of cardiovascular calcification in patients with atrial fibrillation and/or pulmonary embolism (IRIVASC) Trial is aiming to definitively answer this question by including around 200 patients followed up for 2 years.

## 2 METHODS

### 2.1 Trial design

The IRIVASC study is a multicenter, prospective interventional trial randomizing patients with atrial fibrillation or pulmonary embolism requiring long‐term OAT into two different treatment regimens: VKA versus rivaroxaban. VKA dosage was adjusted according to a target INR level of 2.0–3.0. The rivaroxaban dosage was chosen according to official labeling depending on renal function (15 mg once daily for patients with estimated glomerular filtration rate (eGFR) < 30 ml/min/1.73² and 20 mg once daily for patients with eGFR ≥ 30 ml/min/1.73²). The study had an open‐label design. At initiation the study only enrolled patients with a de‐novo indication for OAT; after amendment # 2 (date November 09, 2013) patients with pre‐existing OAT were also approved for inclusion.

Three German study centers participated in the IRIVASC trial: the University Hospital RWTH Aachen (UKA), the Sankt‐Antonius Hospital Eschweiler (SAH), and the Klinikum Coburg. The recruitment was competitive. The randomization was performed 1:1. The treatment was unblinded and INR level controls were done both at the study center during follow‐up visits as well as at the patient's primary care physicians.

### 2.2 STUDY OBJECTIVES

*The primary endpoint* is the *p*rogression of coronary and aortic valve calcification (quantified by the calcification volume score) as assessed by cardiac computed tomography (CT) at 24 months. The secondary objectives can be found in the Supporting Information Material.

### 2.3 ETHICS

The ClinicalTrials.gov Identifier is NCT02066662 and the EudraCT # is 2012‐003768‐28.

The study protocol has been approved by the RWTH Aachen Ethics Committee (UK Aachen EC' EK 239/12). The investigator followed all appropriate requirements, including Good Clinical Practice and Federal Regulation.

### 2.4 Patient recruitment and evaluation

Patients with the following criteria were included in the trial:

(i) Adults with need for long‐term OAT according to current international guidelines for the treatment of atrial fibrillation (American College of Cardiologists (ACC)/American Heart Association (AHA)/European Society of Cardiology (ESC)‐guidelines) and/or pulmonary embolism (ACCP/ESC guidelines).
(ii) Existent coronary or valvular calcification, or both and an Agatston Score (AS) > 50 in at least one location as assessed by MSCT at screening.

The exclusion criteria can be found in the Supporting Information Material.

### 2.5 Conduct of the trial

After evaluation of inclusion and exclusion criteria and after written informed consent the patients were randomized to two arms (1:1):

Arm A: Rivaroxaban (tablet) with 20 mg once daily for patients with atrial fibrillation with eGFR > 49 ml per minute and 15 mg rivaroxaban once daily for patients with eGFR of 15–49 ml. Rivaroxaban (tablet) for patients with pulmonary embolism: 2× a day 15 mg at Days 1–21 and 1 × 20 mg from Day 22.

Arm B: Adjusted dose coumadin/phenprocoumon (tablet) titrated according to target INR with a target range of 2.0–3.0 according to ACC/AHA/ESC guidelines.^2^

To limit the number of study patients and to detect a sufficient progression of coronary artery or aortic valve calcification within the follow‐up period of 2 years, this study was limited to high‐risk patients defined by baseline calcium AS > 50 (either coronary or valvular).

This threshold ensures a measurable progression in outmost patients as the progression of valvular and coronary calcification is dependent on the baseline calcium load (23–25).

### 2.6 Patient follow‐up

Blood collection for serum chemistry, hematology, coagulation, and batch analysis of proteins involved in the process of calcification such as MGP and Fetuin‐A are measured at 1 Week, 1, 3, 6, 9, 12, and 24 months. Echocardiography was performed at baseline, 6, 9, 12, and 24 months (evaluation by an experienced echocardiographer with at least 10 years' experience). Calcification score (coronary or valvular) as assessed by cardiac CT was measured at 12 and 24 months. To improve clinical consistency, all CT scans were evaluated in a consensus reading by an experienced radiologist and an experienced cardiologist. Measurement of changes in intima‐media thickness of carotid artery (IMT) and flow‐mediated vasodilation of brachial artery (FMD) are performed at baseline, 6, 12, and 24 months. Blood pressure was measured at 1 week and at 1, 6, 9, 12, and 24 months. Electrocardiography was performed at 12 and 24 months. Adverse Events were evaluated at 1 week, 1, 6, 9, 12, and 24 months. MACE and major and nonmajor bleedings are documented at every visit.

The study duration was approximately 6.5 years. The planned duration of study inclusion was 55 months; the follow‐up period is 24 months. According to the trial design patients were followed‐up at 1 week as well as 1, 3, 6, 9, 12, and 24 months after inclusion.

**Figure 1:**
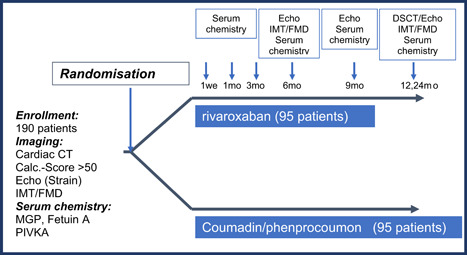
Trial design

The design of the study is illustrated in Figure 1.

### 2.7 Cardiac calcification assessment with CT‐scanning

The progression of valvular and coronary calcification will be assessed by cardiac CT. Valvular calcifications, representatively measured by aortic valve calcification, will be quantified by aortic valve calcium scoring (AVCS), coronary calcifications will be quantified by coronary calcium scoring (CAC), using the AS for CAC and an Agatston‐equivalent score for AVCS, the volume score (expressed as millimeter cubic) and the mass score (expressed as milligram calcium hydroxyapatite), respectively. A previous large interventional trial (ADVANCE) in hemodialysis patients revealed differences in calcification development quantification depending upon the specific score applied.^19^ Thus, the reliability of calcification assessment by each of these three scores needs to be discussed. In summary, the volume calcification score is regarded as superior (most reliable) to the others. AS reliability is sensitive to image noise and slice thickness, which lowers the reproducibility of this method. Furthermore, AS is a virtual value and does not represent a physical value. Although the concept of mass score theoretically seems to be more robust and reliable than volume score and AS,^20^ its calculation is challenging, and hence, the mass score is usually not used in international multicenter trial.^21^ Volume score is less sensitive to image noise, or in other words, to the patient's habitus. Therefore, measurements of the volume score are more reliable and robust,^19^ which goes in line with, for example, the results of the MESA cohort.^22^ Overall, the present study will assess Agatston and volume scores in parallel and therefore provide results of a clinically frequently applied and established score (AS) and a reliable, stable score (volume) at the same time.

### 2.8 MSCT

For calcium scoring, noncontrast ECG‐triggered MSCT of the whole heart was performed in a step‐and‐shoot‐technique, as generally accepted and recommended by current guidelines. Scan length is defined by a scout view from the tracheal bifurcation to the bottom of the heart silhouette. CT scanners at the different study sites are at least 64‐slice MSCT scanners dedicated to cardiac imaging (SOMATOM Definition Flash and Aquilion Prime, Canon Medical Systems). To achieve reproducible and comparable calcium scoring results, every scanner is separately calibrated with a dedicated calcium calibration phantom (Anthropomorphic Cardio CT Phantom, size 300 × 200 × 100 mm, QRM Quality Assurance in Radiology and Medicine GmbH). The same basic scan parameters (e.g., 80 mAs, 120 kV, 3/0.75 mm slice thickness, same rotation time, same collimation, same pitch) are used at all sites and if necessary, are adapted to each participant's individual physique. Afterward, CT scans are transferred to a dedicated postprocessing and measurement workstation (SyngoCaScoring, Wizard; Siemens) for quantification of valvular and coronary calcifications. Both, valvular and coronary calcification measurements are performed using a dedicated software tool (Syngo MMWP, VA13A; Siemens). The volume score is determined by the calculated volume (Hounsfield units > 130 and a minimum size of 0.5 mm^3^) based on isotropic interpolation.^23^ The AS is calculated by multiplying the density and size of the calcified areas as previously described.^24^ According to the above‐listed exclusion criteria, patients with extensive, multivessel coronary stents, which make coronary artery calcification scoring impossible or unreliable, are excluded by study design. In patients with single‐vessel stenting, a CAC is carefully done with manual exclusion of the stent, if technically possible. In case of technical impossibility of CAC measurement, patients are excluded from further study participation.

The core cardiology is located at the RWTH University Hospital radiology department. All measurements are performed by the same radiologist with more than 10 years of experience in calculating and interpreting calcium deposits in coronary arteries and heart valves. The radiologist is blinded to the patients' individual history, treatment randomization, and the date of the examination. To improve intra‐reader reproducibility, baseline and follow‐up CT are presented to the reader immediately following each other in random order.

### 2.9 Statistical analysis

All randomized patients with OAT due to atrial fibrillation or atrial fibrillation and/or pulmonary embolism are included in the analyses. Descriptive analyses of study data include frequencies, mean, standard deviation median, minimum, maximum values, and frequencies of missing data. Furthermore, a description of the change from baseline (absolute difference and percentage change) will be given. The calcification volume score is used in the primary analysis to evaluate the treatment effect. A linear mixed‐effects model is used with treatment effect, visit, baseline measurement, and treatment‐visit interactions as fixed effects and random intercepts for participants are included. For the primary inferential analysis, the hypothesis that the treatment‐visit interaction parameters are jointly equal to zero is tested using an *F *test. Additionally, 95% confidence intervals of parameter estimates are presented. In secondary analyses, the valvular and aortic calcification endpoints are analyzed using similar mixed models as in the primary analysis. An interim analysis is planned after 12 months of follow‐up. Significance levels are adjusted using the Fleming‐Harrington‐O'Brien method. A 5% overall significance level is used in the primary analysis. All statistical analyses are conducted using SAS or SPSS.

For the calculation of sample size for detecting the difference in volume score change between the two treatment groups the standard deviation of the change in volume, score was assumed to be 234 units, based on previous data measuring volume score change over 12 months in placebo‐treated patients with asymptomatic or mildly symptomatic aortic valve calcification.^20^ Given this assumption on the variability of volume score change, a sample size of 95 per treatment group (*n* = 190 altogether) would allow a detection of 96 units difference between the treatment groups with an 80% power using a Type 1 error of 0.05.

## 3 RESULTS

In total 192 patients were recruited between 2013 and 2018. Of these, baseline data is available for 188 patients (95 in the Phenprocoumon group and 93 in the Rivaroxaban group). The median age of the patients was 72 (IQR 67–76) in the Phenprocoumon group and 68 (IQR 65–75) in the Rivaroxaban group. Within the groups, there were 73.6% and 69.9% male patients, respectively. The main indication for anticoagulation was atrial fibrillation (95.8% and 96.8%, respectively) with pulmonary embolism being the only other indication (Table 1). In total, data for 164 patients (82 patients per group [85%]) is available at 12 months follow up and for 91 patients (45 in the Rivaroxaban group [47%] and 46 [48%] in the Phenprocoumon group). We expect the results of the study to be ready for publication by the end of 2022.

**Table 1:** Characteristics of the patients at baseline (IRIVASC)

	Phenprocoumon (*N *= 95)	Rivaroxaban (*N* = 93)
Median age (IQR), year	72 (67–76)	68 (65–75)
Male, no. (%)	70 (73.6)	65 (69.9)
Diabetes mellitus, no. (%)	23 (24.7)	26 (29.2)
Smoker, no. (%)	11 (11.5)	14 (15)
Non smoker, no. (%)	39 (41)	45 (48.3)
Ex. smoker, no. (%)	42 (44.2)	28 (30.1)
Previous MI, no. (%)	25 (26.3)	26 (27.9)
Previous stroke, no. (%)	16 (16.8)	8 (8.6)
Coronary heart disease (CHD), no. (%)	51 (54.8)	54 (60.6)
Median systolic blood pressure, right (IQR), mmHg	130 (120–143,7)	135,5 (122,7–149)
Median systolic blood pressure, left (IQR), mmHg	130 (120–140)	134 (124–145)
Median total cholesterol (IQR), mg/dl	173 (149.7–204.2)	176 (142–214.5)
Median LDL‐cholesterol (IQR), mg/dl	109 (85.5–128,5)	102.5 (82–138)
Median GFR (IQR), ml/min/1.73 m^2^	65.6 (52.8—81.5)	72.3 (57.2–84.7)
Median hemoglobin (IQR), g/dl	13.9 (13.2–15.2)	14.2 (13.1–15)
Median CHA2DS2VASc score (IQR)	3 (3–4)	3 (2–4)
Indication for anticoagulation:		
1. Atrial fibrillation, no. (%)	91 (95.8)	90 (96.8)
2. Pulmonary embolism, no. (%)	4 (4.2)	3 (3.2)
Type of atrial fibrillation		
1. Paroxysmal, no. (%)	51 (56)	57 (63.3)
2. Persistent, no. (%)	15 (16.5)	14 (15.6)
3. Permanent, no. (%)	16 (17.6)	15 (16.7)
4. New onset, no. (%)	1 (1.1)	1 (1.1)
5. Not further classified, no. (%)	8 (8.8)	3 (3.3)

### 3.1 Study limitations

This study has some limitations. Owing to the different mechanism of action of both drugs, the trial is open‐label. Furthermore, to increase recruitment, the study enrolled and randomized patients currently on OAT. It could have been better to only enroll OAT naïve patients because the difference in duration of OAT therapy before randomization could become a source of bias. Finally, the study only includes patients with a baseline AS of >50 to limit the number of study patients and to detect a sufficient progression of coronary artery or aortic valve calcification within the set follow‐up period, thus potentially limiting the ability to detect smaller differences between the groups.

## 4 DISCUSSION

Numerous studies indicate that the presence of vascular calcification is an independent predictor of the future risk for cardiovascular events and mortality. Despite local differences at different anatomic sites, such cardiovascular calcifications have important implications for future cardiovascular events.^25^ Generally, the presence of vascular calcification associated with an increased risk for adverse coronary events,^26^ and moreover, patients with moderate to severe calcification have an unfavorable prognosis compared to patients with no or mild calcification.^26^, ^27^, ^28^ Hence, iatrogenic induction of vascular calcification as a potential side effect of chronic VKA treatment is of utmost importance.^26^

With vitamin K being necessary for MGP activation two important open issues emerge with potentially direct impact upon human health: First, does vitamin K replenishment reduce the occurrence and/or progression of vascular calcification? Second, should NOACs be the preferable OAT compared to VKA also because they are neutral in terms of vascular calcification induction?

Regarding the first issue, several experimental and epidemiological lines of evidence clearly point to protective effects of vitamin K in the cardiovascular system: The Rotterdam study revealed the existence of an inverse association between vitamin K intake (menaquinone) with aortic calcification and coronary heart disease in humans.^29^ Our research group was able to show that serum uncarboxylated MGP may be used as a biomarker to identify those at risk for developing cardiovascular calcification using a competitive enzyme‐linked immunoassay (ELISA) which specifically recognizes uncarboxylated MGP.^30^ Vitamin K replenishment could prevent further calcifications in a rat model of vascular calcifications.^31^ Moreover, we could show in a small randomized, interventional prospective pilot trial, that 2000 mg of vitamin K (phytomenadione) per day over 1 year could slow down the progression of aortic valve calcification in patients with mild to moderate calcific aortic stenosis.^11^ An ongoing prospective study is currently investigating this finding in a larger cohort of aortic stenosis patients.^32^ J. Floege is leading a trial investigating if vitamin K replenishment can slow down the progression of coronary artery calcification in hemodialysis patients—a group of patients with specific high cardiovascular calcification pressure.^33^

The basic hypothesis of the IRIVASC trial is that the former standard anticoagulant treatment with VKA is associated with accelerated coronary or valvular calcification as assessed by cardiac CT. Since NOACs do not interfere with vitamin K metabolism such pro‐calcifying effects of VKA should be absent in NOAC‐treated patients. This potential neutrality in terms of cardiovascular calcification induction might translate into already established as well as novel, yet undetermined clinical benefits for NOACs. Indeed, it has been shown that NOACS induce a lower rate of intracranial hemorrhage. A potential reason for this finding might be the preservation of vascular wall integrity and elasticity in the absence of calcification induction.

Testing in vivo the hypothesis of a deleterious calcification effect of warfarin/phenprocoumon‐treatment in contrast to rivaroxaban‐treatment is likely to have a significant impact on the clinical decision‐making about the mode of OAT in cardiovascular high‐risk patients. Specifically, patient groups with underlying systemic pro‐calcification disorders such as diabetes and chronic kidney disease might benefit from avoiding additional pro‐calcification actions of VKA. However, up to date, there is no data about the progression of coronary and valvular calcification during the treatment with rivaroxaban compared to the standard OAT with warfarin from a randomized controlled trial. Hence, we initiated the IRIVASC trial to close this essential knowledge gap.

## Supporting information

Supporting information.

## Data Availability

The data that support the findings of this study are available on request from the corresponding author. The data are not publicly available due to privacy or ethical restrictions.
