# Supplementary material for: Influence of rivaroxaban compared to vitamin K antagonist treatment upon development of cardiovascular calcification in patients with atrial fibrillation and/or pulmonary embolism
Source: Clin Cardiol. 2022 Mar 25;45(4):352–8. doi: 10.1002/clc.23819 (PMC9019879; doi:10.1002/clc.23819)
Supplement: Supplementary file 1 — Supporting information. [file CLC-45-352-s001.docx]

**Secondary outcome variables:**

- Progression of coronary and valvular calcification (Agatston and calcification mass score)
- Progression of aortic calcification (aortic Agatston Score)
- Changes in intima-media thickness of carotid artery (IMT) and flow-mediated vasodilation of brachial artery (FMD)
- Serum chemistry including matrix Gla-protein level changes and fetuin-A (baseline/ follow up)
- Changes in ventricular diastolic function parameters as determined by echocardiography (strain/strain-rate imaging)
- Occurrence of major cardiovascular complications (MACE)

**Exclusion Criteria**

Patients who meet any one of the following exclusion criteria are not included in the trial:

1. Patients with previous coronary stent implantation in a way which makes coronary artery calcification scoring impossible or have unreliable and absence of valvular calcification with Agatston score > 50
2. Chronic kidney disease (CKD) Stage 5 (eGFR <15 mL/min/1.73²)
3. Liver disease with coagulopathy or other bleeding disorders including cirrhotic patients with Child Pugh B and C
4. Clinically significant active bleeding
5. Reasons not allowing free randomization between the two anticoagulant regimen including prosthetic heart valves.
